# Neoadjuvant capecitabine, radiotherapy, and bevacizumab (CRAB) in locally advanced rectal cancer: results of an open-label phase II study

**DOI:** 10.1186/1748-717X-6-105

**Published:** 2011-08-31

**Authors:** Vaneja Velenik, Janja Ocvirk, Maja Music, Matej Bracko, Franc Anderluh, Irena Oblak, Ibrahim Edhemovic, Erik Brecelj, Mateja Kropivnik, Mirko Omejc

**Affiliations:** 1 Institute of Oncology, Zaloska 2, 1000 Ljubljana, Slovenia; 2 University Medical Centre, Zaloska 7, 1000 Ljubljana, Slovenia

**Keywords:** capecitabine, chemoradiation, bevacizumab, locally advanced rectal cancer, LARC, phase II study

## Abstract

**Background:**

Preoperative capecitabine-based chemoradiation is a standard treatment for locally advanced rectal cancer (LARC). Here, we explored the safety and efficacy of the addition of bevacizumab to capecitabine and concurrent radiotherapy for LARC.

**Methods:**

Patients with MRI-confirmed stage II/III rectal cancer received bevacizumab 5 mg/kg i.v. 2 weeks prior to neoadjuvant chemoradiotherapy followed by bevacizumab 5 mg/kg on Days 1, 15 and 29, capecitabine 825 mg/m^2 ^twice daily on Days 1-38, and concurrent radiotherapy 50.4 Gy (1.8 Gy/day, 5 days/week for 5 weeks + three 1.8 Gy/day), starting on Day 1. Total mesorectal excision was scheduled 6-8 weeks after completion of chemoradiotherapy. Tumour regression grades (TRG) were evaluated on surgical specimens according to Dworak. The primary endpoint was pathological complete response (pCR).

**Results:**

61 patients were enrolled (median age 60 years [range 31-80], 64% male). Twelve patients (19.7%) had T3N0 tumours, 1 patient T2N1, 19 patients (31.1%) T3N1, 2 patients (3.3%) T2N2, 22 patients (36.1%) T3N2 and 5 patients (8.2%) T4N2. Median tumour distance from the anal verge was 6 cm (range 0-11). Grade 3 adverse events included dermatitis (n = 6, 9.8%), proteinuria (n = 4, 6.5%) and leucocytopenia (n = 3, 4.9%). Radical resection was achieved in 57 patients (95%), and 42 patients (70%) underwent sphincter-preserving surgery. TRG 4 (pCR) was recorded in 8 patients (13.3%) and TRG 3 in 9 patients (15.0%). T-, N- and overall downstaging rates were 45.2%, 73.8%, and 73.8%, respectively.

**Conclusions:**

This study demonstrates the feasibility of preoperative chemoradiotherapy with bevacizumab and capecitabine. The observed adverse events of neoadjuvant treatment are comparable with those previously reported, but the pCR rate was lower.

## Introduction

Treatment of locally advanced rectal cancer (LARC) is multimodal and generally consists of surgery, radiation and chemotherapy. Preoperative radiotherapy (RT) has been investigated as a neoadjuvant treatment for rectal cancer to improve local control and survival rates. The potential advantages of preoperative RT include decreased tumour spread (local and distant), reduced acute toxicity, increased sensitivity to RT and enhanced sphincter preservation during surgery [1-4]. In LARC, the addition of 5-fluorouracil (5-FU) to preoperative RT has been shown to improve pathological complete response rate, tumour downstaging [5] and locoregional control [6,7] compared with RT alone. Furthermore, preoperative chemoradiotherapy improves locoregional control with less toxicity compared with postoperative chemoradiotherapy [4]. Thus, preoperative chemoradiotherapy with continuous infusional 5-FU has become a standard of care in rectal cancer, especially in tumours of the lower and middle rectum.

The oral fluoropyrimidine capecitabine was designed to mimic continuous 5-FU infusion and to generate 5-FU preferentially in tumour tissue. Capecitabine has demonstrated efficacy comparable with intravenous 5-FU in metastatic colorectal cancer as well as in the adjuvant setting in colon cancers [8-14]. Furthermore, capecitabine has been investigated in various protocols for rectal and other gastrointestinal cancers in combination with RT [15]; indeed, equivalence of capecitabine plus RT and 5-FU plus RT as preoperative therapy in LARC was demonstrated in the systematic review by Saif and colleagues [16]. Recently, two phase III trials, the large National Surgical Adjuvant Breast and Bowel Project (NSABP) R-04 Intergroup study [17] and a German trial [18], have confirmed that capecitabine is non-inferior to 5-FU as component of neoadjuvant radiochemotherapy in rectal cancer, and a retrospective analysis from a single centre found preoperative capecitabine plus RT to have more favourable results and higher downstaging rates that infusional 5-FU plus RT [19]. Preoperative capecitabine-based chemoradiation is now a standard treatment for LARC [4].

Phase II studies evaluating preoperative doublet chemotherapy of oxaliplatin or irinotecan plus 5-FU or capecitabine combined with concurrent radiotherapy in LARC have reported either no change or an increase in pathological complete response with the addition of oxaliplatin or irinotecan, and this addition also frequently resulted in increased acute toxicity [17,18,20-26].

The addition of bevacizumab, a humanized monoclonal antibody to vascular endothelial growth factor (VEGF), to chemotherapy has been shown to increase the efficacy of therapy in metastatic colorectal cancer [27]. It is postulated that combining bevacizumab with chemoradiation may increase antitumour efficacy by maximizing inhibition of the VEGF pathway [28,29]. That said, there are relatively limited data on the safety and efficacy of bevacizumab in combination with chemotherapy and radiation in the neoadjuvant setting [30-34]. In this study we explored the safety and efficacy of neoadjuvant capecitabine, concurrent radiotherapy and bevacizumab (CRAB) in LARC.

## Patients and Methods

We undertook a prospective, open-label, single-arm phase II study in patients with histologically proven adenocarcinoma of the rectum (Clinicaltrials.gov registration number: NCT00842686). The study was approved by the relevant institutional review board, the National Ethics Committee and the Ministry of Health. All patients gave written informed consent prior to any study procedure.

### Patients

Patient pretreatment work-up comprised a complete history, physical examination, full blood count, serum biochemistry, carcinoembryonic antigen, chest radiography, ultrasonography and/or computed tomography (CT) scan of the whole abdomen. The extent of locoregional disease was determined by magnetic resonance imaging (MRI) of the pelvis of each patient. Eligible patients had to have a histologically verified stage II or III adenocarcinoma of the rectum, the disease had be considered either resectable at the time of entry or thought likely to become resectable after preoperative chemoradiation with no evidence of distant metastases. Other key inclusion criteria were: age 18-80 years; World Health Organization performance status of 0-2; adequate bone marrow, liver, renal and cardiac function (no history of clinically significant cardiovascular disease); no prior radiotherapy, chemotherapy or any targeting therapy for rectal cancer; ability to swallow oral medications; and signed informed consent. Key exclusion criteria included: other co-existing malignancy or malignancy within the last 5 years prior the enrolment other than non-melanoma skin cancer or in situ carcinoma of the cervix; patients with severe concurrent medical or psychiatric illness; a known hypersensitivity to study drug; and pregnant or lactating patients.

### Study design and treatment

**Figure 1:**
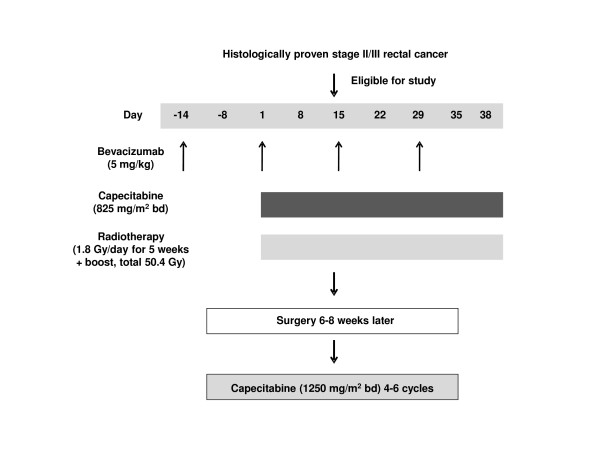
**Study design and treatment schedule**.

The study design and treatment schedule are shown in Figure 1. Three-dimensional CT-based treatment planning was performed. The CT was taken on treatment position with 5 mm thick slices. The clinical target volume was defined as covering the small pelvis from the L5-S1 interspace to 5 cm below the primary tumour. The lateral borders were 5 mm outside the true bony pelvis. The posterior margin covered the sacrum and the anterior margin encompassed the posterior one-third to one-half of the bladder and/or vagina. An additional 1 cm in all directions was added to the clinical target volume to obtain the planning target volume. The dose was prescribed to cover the planning target volume with a 95% reference isodose (95% of the International Commission on Radiation Unit point dose).

RT was initiated on Day 1. Patients received a total irradiation dose of 45 Gy to the pelvis plus 5.4 Gy as a boost to the primary tumour in 1.8 Gy daily fractions over 5.5 weeks. Radiotherapy was delivered using 15 MV photon beams and four-field box technique, once daily, 5 days per week. All fields were treated daily. Multileaf collimators were used to shape individual radiation fields and for the protection of normal tissues. Patients were irradiated in a prone position with a full bladder and using a belly board to minimize exposure of the small bowel.

Chemotherapy was administered concomitantly with RT and consisted of oral capecitabine at a daily dose of 1650 mg/m^2^, divided into two equal doses given 12 hours apart. One dose was taken 1 hour prior to RT. The chemotherapy started on the first day of RT (Day 1), finished on the last day of RT (Day 38) and was continuous throughout the RT period (i.e. it included weekends). Bevacizumab was administered at a dose of 5 mg/kg on treatment days: -14, 1, 15 and 29. The drug was delivered as an intravenous infusion over a 30-90-min period.

Resection was performed 6-8 weeks after the completion of chemoradiotherapy. A total mesorectal excision was the recommended operation for mid and distal rectal tumours. Surgical management included a sphincter-preservation approach whenever possible. The option for a temporary colostomy during surgery was left to the surgeon's discretion. Complications after surgery were recorded.

In patients achieving histopathological R0 or R1 resection, adjuvant chemotherapy was recommended: this comprised capecitabine 1250 mg/m^2 ^orally twice daily on Days 1-14 every 3 weeks; 4 (R0 resection) or 6 cycles (R1 resection) were recommended, beginning 6-8 weeks after surgery.

### Study assessments

It has been shown that complete eradication of the primary tumour observed in the histopathological specimen (pathological complete response [pCR]) correlates with a favourable overall prognosis, so obtaining a pCR might be beneficial [35]. Thus, the primary endpoint of pCR rate was selected for the current analysis. Secondary endpoints were: pathological response rate (plus tumour regression grade [TRG] according to Dworak scale); rate of sphincter-sparing surgical procedure; histopathological R0 resection rate; acute and late toxicity (SOMA/LENT scale); locoregional failure rate; disease-free survival; and overall survival. The effect of preoperative chemoradiotherapy on tumour downstaging was assessed by comparing the pretreatment radiologically determined TNM stage with the postoperative pathologic TNM stage. As an exploratory objective, the mutation status of KRAS in pre-therapeutic biopsies was established and correlation to pathological response was assessed.

During treatment, patients were evaluated weekly. Clinical examinations, complete blood count and serum chemistry analysis were performed. Adverse events were assessed according to National Cancer Institute Common Toxicity Criteria (NCI-CTC) version 3.0. Re-evaluation of the primary tumour with pelvic MRI was performed four weeks after the completion of preoperative treatment.

Postoperative, pathological evaluation of the surgical specimen was performed. pCR was defined as the complete disappearance of all tumour cells. Histological regression of the primary tumour was semi-quantitatively determined according to a 5-point TRG scale: TRG 0, no regression; TRG 1, minimal regression (dominant tumour mass with obvious fibrosis and/or vasculopathy); TRG 2, moderate regression (predominantly fibrotic changes with few tumour cells or groups); TRG 3, good regression (very few tumour cells in fibrotic tissue); TRG 4, total regression (no tumour cells, only fibrotic mass).

Follow-up visits were scheduled at 3, 6, 12, 18, 24, 36, 48 and 60 months following the end of adjuvant chemotherapy.

### Statistical analysis

The primary endpoint of the study was pCR rate. In the medical literature, phase II studies of capecitabine and RT suggest a pCR rate in the range of 4-31%, while in our published study the pCR rate was approximately 9%. We aimed to evaluate whether a 23% pCR rate could be achieved by adding bevacizumab to standard preoperative treatment. Setting 10% as the lowest pCR rate of interest, and with alpha error of 5% and power of 80%, at least 50 evaluable patients were needed (calculated using power sample calculation, for two samples, percentages, α = 5%, 1-β = 20%). Assuming that ≥10% of patients would not be evaluable, the estimated sample size required was at least 60 patients.

Statistics were descriptive and all data were analysed using the SPSS statistical software package, version 13 (SPSS Inc., Chicago, IL, USA).

## Results

### Patient characteristics and treatment rates

Between February 2009 and March 2010, a total of 61 patients were recruited. Patients' baseline and disease characteristics are summarized in Table 1. Three patients (4.9%) presented with stage T2 disease, 53 (86.9%) with stage T3, 5 (8.2%) with stage T4; lymph node involvement was detected in 49 patients (80.3%). The most frequent MRI staging was uT3N+ (67%). In 28 patients (45.9%) the tumour invaded the mesorectal fascia and in half of the patients (50%) the primary tumour was sited ≤5 cm from the anal verge.

**Table 1:** Patients' baseline and disease characteristics

Characteristics	Patients (n = 61)
Median age, years (range)	60 (31-80)
Gender, n (%):	
Male	39 (64)
Female	22 (36)
WHO performance status, n (%)	
0	52 (85)
1	9 (15)
TN clinical stage, n (%)	
T3N0	12 (19.7)
T2N1	1 (1.7)
T3N1	19 (31.1)
T2N2	2 (3.3)
T3N2	22 (36.1)
T4N2	5 (8.2)
Median clinical tumour size per MRI, cm (range)	6 (1-12)
Median tumour distance from anal verge, cm (range)	6 (0-11)
Type of surgery^a^, n (%)	
Low anterior resection	35 (57.4)
Coloanal reconstruction	10 (16.4)
Abdominoperineal resection	14 (23.0)
Pelvic exenteration	2 (3.3)

^a^As planned before the start of preoperative chemoradiotherapy.

MRI, magnetic nuclear imaging; N, node; T, tumour; WHO, World Health Organization.

All patients received neoadjuvant chemoradiotherapy plus bevacizumab. Treatment was terminated in one patient as a result of withdrawal of informed consent following four weeks of treatment. All other patients received 100% of the expected radiation treatment. Treatment interruption was necessary for 7 patients (11.6%) because of grade 2 (n = 2) and grade 3 (n = 3) leucopenia, grade 3 diarrhoea (n = 1), and grade 3 (n = 1) and grade 4 (n = 1) vascular toxicity. Other grade 3 toxicities included dermatitis (n = 6), proteinuria (n = 4) and hypertension (n = 1). There were no treatment-related deaths during the study.

RT was interrupted for 2-7 days as a result of treatment (median interruption: 2 days), while 56 patients (91%) received 95-100% of the designated chemotherapy dose. Overall, 58 patients (95.1%) received all four infusions of bevacizumab while the remaining 3 patients received three infusions.

### Treatment-related toxicities

The frequency and grade of treatment-related acute toxicities are summarised in Table 2. The most frequent adverse event reported with chemoradiotherapy was grade 2 and 3 radiodermatitis. During treatment, 25 patients lost weight; the maximum body weight loss was 6.5% (median 3.3%). Of the remaining patients, 26 maintained a constant weight and nine experienced a weight increase of up to 5% (median: 2.4%).

**Table 2:** Acute toxicities occurring during preoperative chemoradiotherapy

	Patients, n (%)			
	
Toxicity	Grade 1	Grade 2	Grade 3	Grade 4
Haematological:				
Leucocytopenia	12 (19.7)	5 (8.2)	3 (4.9)	-
Anaemia	5 (8.2)	-	-	-
Non-haematological:				
Diarrhoea	14 (22.9)	4 (6.5)	1 (1.6)	-
Fatigue	7 (11.5)	3 (4.9)	-	-
Nausea	5 (8.2)	-	-	-
Anorexia	2 (3.3)	-	-	-
Dermatitis	3 (4.9)	14 (22.9)	6 (9.8)	-
Hand-food syndrome	5 (8.2)	2 (3.3)	-	-
Cystitis	3 (4.9)	-	-	-
Hepatotoxicity	2 (3.3)	2 (3.3)	-	-
Vascular	-	-	1 (1.6)	1 (1.6)
Proteinuria	10 (16.4)	2 (3.3)	4 (6.5)	-
Hypertension	2 (3.3)	2 (3.3)	1 (1.6)	-
Infection	3 (4.9)	5 (8.2)	-	-
Pain	20 (32.8)	3 (4.9)	-	-
Bleeding	10 (16.4)	-	-	-

According to National Cancer Institute Common Toxicity Criteria (version 3)

### Surgery rates and outcomes

All patients underwent definitive surgery, although one patient revealed distant metastases after completion of chemoradiotherapy. Surgery was performed 25 to 79 days (median: 55 days) after the last day of chemoradiotherapy. Exploratory surgery was performed in only 1 patient because of a large, unresectable T4 tumour with peritoneal carcinomatosis. The median hospital stay for surgery was 11 days (range: 7-32 days).

Radical resection was achieved in 57 patients (95%) and 42 patients (70%) underwent sphincter-preserving surgery. A temporary stoma was created in 41 patients. In one patient pathohistological examination of the surgical specimen revealed malignant melanoma; this patient was considered misdiagnosed and excluded from the efficacy analysis.

Pathological TNM stages in relation to preoperative TNM status are presented in Table 3. TRG 4 (pCR) was recorded in 8/60 patients (13.3%) and TRG 3 in 9/60 patients (15.0%). T-, N- and overall downstaging rates were 46.7%, 65.0% and 75.0%, respectively.

**Table 3:** Distribution of postoperative pathological TMN stages compared with pretreatment clinical stages (n = 60)

Before	After surgery (pTNM)							
	
	T0N0	T1N0	T2N0	T3N0	T2N1	T3N1	T4N1	T3N2
**T3N0**	3		4	3		2		

**T2N1**			1					

**T3N1**	5	3	4	4	1	1		

**T2N2**			1					1

**T3N2**		1	2	13		1	1	4

**T4N2**		1	2	1		1		

**Total**	8 (13.3%)	5 (8.3%)	14 (23.3%)	21 (35%)	1 (1.7%)	5 (8.3%)	1 (1.7%)	5 (8.3%)

c - Clinical, p - pathological, T - Tumour, N - Node, M - Metastasis.

KRAS mutations were found in 20 (33.9%) out of 59 bioptic tumour samples obtained before preoperative treatment. *KRAS *status was not associated with pathological response.

In total, 38 patients (62.3%) developed perioperative complications. The most frequent were delayed wound healing (n = 18, 30.0%), infection/abscess (n = 12, 20.0%) and anastomotic leakage (n = 7, 11.7%). Six patients required surgical re-intervention for anastomotic leakage (n = 3), abdominal abscess (n = 2) and pneumothorax (n = 1). There were no perioperative deaths. A summary of perioperative toxicity is shown in Table 4.

**Table 4:** Perioperative adverse events (n = 60)

Complication	Patients, n (%)^a^
Delayed healing of postoperative wound	18 (30.0)
Infection/abscess	12 (20.0)
Pneumothorax	1 (1.7)
Anastomotic leakage	7 (11.7)

^a^Patients could have more than one adverse event.

Postoperative chemotherapy was administered to 51 (83.6%) patients. Reasons for not administering adjuvant chemotherapy were: progression of the disease (n = 2), misdiagnosis (n = 1); withdrawal from study (n = 1); > 8 week interval between the operation and adjuvant therapy (n = 1); and postoperative complications (n = 5). Postoperative chemotherapy comprised capecitabine 1250 mg/m^2 ^on Days 1-14 every 3 weeks for 4 or 6 cycles. A total of 42 patients received all planned cycles. Two patients only received 3 cycles because of disease progression (n = 1) and death because of pulmonary thromboembolism (n = 1); 2 patients only received 2 cycles because of diarrhoea and dehydration (n = 1) and nonspecific chest pain (n = 1); and 3 patients only received 1 cycle because of the development of presacral abscesses (n = 2) and nonspecific chest pain (n = 1).

## Discussion

This phase II study demonstrates the feasibility of preoperative chemoradiation with bevacizumab and capecitabine in patients with LARC. Indeed, a high R0 resection rate was achieved despite tumour invasion of the mesorectal fascia in 46% of patients. A well-accepted approach in the management of LARC is neoadjuvant fluoropyrimidine-based chemoradiation and a number of prospective and retrospective trials have suggested that preoperative capecitabine is at least equivalent to infusional 5-fluorouracil when combined with RT [16-19], and may improve tumour downstaging. In 2009, the US National Comprehensive Cancer Network recommended capecitabine as an acceptable alternative to 5-FU in this setting [36].

The pCR rate of 13% observed with neoadjuvant capecitabine plus bevacizumab plus RT was similar to an earlier phase II study by our group examining neoadjuvant single-agent capecitabine plus RT in LARC [37]. This pCR rate, albeit relatively low, is within the range 0-31% reported across a number of phase II studies evaluating single-agent capecitabine plus RT [38-43]. In one of the largest studies performed to date, the pCR rate was 12% [44], and in the recently presented NSABP-04 study the pCR ranged from 18 to 22% with capecitabine and 5-FU achieving similar rates of improvement but no additional benefit being observed with the addition of oxaliplatin to either of these agents [17]. A study by Ofner and co-workers [45] evaluating preoperative capecitabine and oxaliplatin reported a pCR rate of 10% while studies investigating preoperative capecitabine, oxaliplatin and bevacizumab found rates of 24-36% [32-34].

In the phase II trial by Crane and coworkers [31], 25 patients with LARC received neoadjuvant chemoradiotherapy with bevacizumab (three doses of 5 mg/kg given every 2 weeks) and capecitabine (900 mg/m^2 ^orally twice daily on days of radiation therapy), followed by surgical resection a median of 7.3 weeks later. These authors reported a pCR rate of 32% (8 patients), which is considerably higher than that reported here. One possible explanation for the relatively low pCR rate observed in our study was that the patients in this study had advanced tumours; indeed, most of the patients had regionally advanced disease and in almost half of the patients the tumour had invaded the mesorectal fascia. However, caution is needed when comparing pCR rates as the pCR rate itself is highly dependent on the quality of the pathological examination [46] and a longer interval between end of chemoradiotherapy and surgery (6-8 weeks vs. 2 weeks) has been reported to increase pCR rate without reducing local recurrence rate or survival [47,48]. While there has been much debate about whether pCR is associated with a favourable long-term outcome, a recently published pooled analysis of data from 3105 patients from 14 studies would suggest that patients with pCR after chemoradiation have better long-term outcome than those without pCR [49].

The adverse event profile observed during neoadjuvant capecitabine plus bevacizumab chemoradiotherapy was comparable with those reported in an earlier study involving capecitabine plus bevacizumab with concurrent RT [31]. The most frequent preoperative adverse events were dermatitis, pain and leucopenia, and adverse events related to bevacizumab therapy were relatively infrequent. Any postoperative adverse events were mainly related to delayed wound healing and infection/abscess. No treatment-related deaths were recorded. These results, together with those of Crane and co-workers [31] suggest that the combination of neoadjuvant capecitabine plus bevacizumab with concurrent RT is feasible and well tolerated in the treatment of LARC. A high radical resection rate suggests its potential positive effect on tumour downstaging. The observed adverse events during neoadjuvant treatment in our study are comparable with those reported previously; however, no clinically relevant increase in pathologic response rate was observed. Longer follow-up is needed to assess the impact on other efficacy endpoints.

Long-term follow-up data on survival and local control in patients with LARC having undergone neoadjuvant capecitabine plus bevacizumab chemoradiotherapy followed by surgery are eagerly awaited. It will also be interesting to compare any long-term follow-up data with that which is currently available at the moment on single-agent capecitabine-based chemoradiotherapy [39,50] to help determine the benefits of adding bevacizumab to the regimen.

## Conclusion

The results of this phase II study indicate that neoadjuvant capecitabine chemoradiotherapy is an effective treatment for patients with LARC and the incorporation of bevacizumab into a standard capecitabine-based chemoradiotherapy regimen is feasible and well tolerated.

## List of abbreviations

5-FU: 5-fluorouracil; CT: computed tomography; LARC: locally advanced rectal cancer; MRI: magnetic resonance imaging; pCR: pathological complete response; RT: radiotherapy; TRG: tumour regression grade; VEGF: vascular endothelial growth factor.

## Competing interests

This was an investigator-initiated trial supported by Roche. The authors declare that they have no financial or non-financial competing interests.

## Authors' contributions

VV: contributions to conception and design, acquisition of data, analysis and interpretation of data; involvement in drafting and reviewing the manuscript. JO: contribution to acquisition of data, analysis and interpretation of data. MM: contribution to acquisition of data, analysis and interpretation of data. MB: contribution to acquisition of data. FA: contribution to acquisition of data, analysis and interpretation of data. IO: contribution to acquisition of data.

IE: contribution to acquisition of data, analysis and interpretation of data. EB: contribution to acquisition of data, analysis and interpretation of data. MK: contribution to acquisition of data. MO: contributions to acquisition of data, analysis and interpretation of data; involvement in drafting and reviewing the manuscript. All authors have read and approved the final version of the manuscript.
